# Association between noninvasive assessment of liver fibrosis and coronary artery calcification progression in patients with nonalcoholic fatty liver disease

**DOI:** 10.1038/s41598-020-75266-4

**Published:** 2020-10-27

**Authors:** Jiwoo Lee, Hwi Seung Kim, Yun Kyung Cho, Eun Hee Kim, Min Jung Lee, In Yong Bae, Chang Hee Jung, Joong-Yeol Park, Hong-Kyu Kim, Woo Je Lee

**Affiliations:** 1 Department of Internal Medicine, Asan Medical Center, University of Ulsan College of Medicine, 88, Olympic-ro 43-gil, Songpa-gu, Seoul, 05505 Republic of Korea; 2 Department of Internal Medicine, Hallym University Sacred Heart Hospital, Hallym University College of Medicine, Anyang, Republic of Korea; 3 Department of Health Screening and Promotion Center, Asan Medical Center, University of Ulsan College of Medicine, 88, Olympic-ro 43-gil, Songpa-gu, Seoul, 05505 Republic of Korea

**Keywords:** Liver fibrosis, Non-alcoholic fatty liver disease, Calcification

## Abstract

Advanced liver fibrosis and coronary artery calcification (CAC) progression has been reported to correlate with cardiovascular disease. This study investigated the association between noninvasive liver fibrosis score and CAC progression in patients with nonalcoholic fatty liver disease (NAFLD). We included 1173 asymptomatic adults with CAC scores from 2007–2013. CAC progression was defined as newly incident CAC or a ≥ 2.5-unit increase in the final CAC score square root. Liver fibrosis was assessed using fibrosis-4 index (FIB-4) score and NAFLD fibrosis score (NFS). A total of 293 (25.0%) subjects developed CAC. Mean baseline FIB-4 score was significantly higher in subjects with CAC. CAC progressed in 20.5% of subjects without NAFLD, 27.5% of those with NAFLD and low FIB-4 scores, and 35.9% of those with NAFLD and intermediate/high FIB-4 scores. On multivariate logistic regression analysis, the odds ratio for CAC progression was 1.70 (95% confidence interval, 1.12–2.58) for subjects with NAFLD plus intermediate/high FIB-4 scores versus those without NAFLD. In the sensitivity analysis, the odds ratio for CAC progression was 1.57 (95% confidence interval, 1.02–2.44) for subjects with NAFLD plus an intermediate/high NFS versus those without NAFLD. Advanced liver fibrosis stage assessed using noninvasive markers is associated with a higher risk of CAC progression in subjects with NAFLD.

## Introduction

Nonalcoholic fatty liver disease (NAFLD) is the most frequent cause of liver disease globally^1^. NAFLD comprises a wide range of conditions, including simple fatty liver, nonalcoholic steatohepatitis (NASH), liver cirrhosis, and hepatocellular carcinoma^1^. In addition to being a liver-related disease, NAFLD is considered an important risk factor for type 2 diabetes mellitus (T2DM), metabolic syndrome, and cardiovascular disease (CVD)^2^. There is increasing evidence that NAFLD is associated with CVD comorbidities such as ischemia-related cardiac disease, cardiomyopathy, and atrial fibrillation^3,4^. Therefore, interest has grown significantly regarding the relationship between NAFLD and CVD.

Liver fibrosis with NAFLD has been deemed a major prognostic factor for mortality and liver-related morbidity^5^. Furthermore, NAFLD accompanied by advanced liver fibrosis reportedly contributes to CVD^6^. The liver fibrosis score evaluated with a noninvasive fibrosis marker in subjects with NAFLD is reportedly associated with the coronary artery calcification (CAC) score^7,8^. Although liver biopsy is the gold standard for evaluating fibrosis degrees in subjects with NAFLD, it is invasive, costly, and prone to complications and sampling errors^9^. Therefore, noninvasive liver fibrosis scoring systems based on clinical data have been used to determine liver fibrosis severity in subjects with NAFLD^10^. These blood-based noninvasive fibrosis scoring systems, including the fibrosis-4 index (FIB-4) score and the NAFLD fibrosis score (NFS), were suggested to have high negative predictive values, thereby preventing unnecessary liver biopsy^11–13^.

The CAC score, measured by multi-detector computed tomography (MDCT), reflects overall coronary artery plaque burden and is used to predict future coronary events and progression^14^. Moreover, the CAC score progression is significantly related to future CVD risk and all-cause mortality^15,16^. Because atherosclerosis has a dynamic series of action, CAC progression is a preferred predictor for development of atherosclerosis, future CVD events and patient prognosis than baseline CAC score^16^.

A few studies to date have reported that advanced liver fibrosis score is associated with CAC^7,8^. However, these studies were cross-sectionally designed to evaluate liver fibrosis score and CAC, preventing the assessment of the relationship between exposure and outcomes. Therefore, the present study evaluated the association between liver fibrosis degree determined by a noninvasive biomarker and CAC progression.

## Results

### Baseline characteristics of the study population

The baseline characteristics of the 1173 study subjects (mean age, 54.1 ± 7.4 years) are presented in Table 1. The subjects were categorized into three sub-groups based on the presence or absence of NAFLD and liver fibrosis severity determined by the FIB-4 score. Of the total cohort, 629 (53.6%) were non-NAFLD, 374 (31.9%) had NAFLD and a low FIB-4 score, and 170 (14.5%) had NAFLD and an intermediate/high FIB-4 score. Overall, male sex was predominant (81.5%). Compared with subjects without NAFLD, those with NAFLD had a higher body mass index (BMI), waist circumference, systolic blood pressure (BP), and diastolic BP; higher serum concentrations of fasting plasma glucose (FPG), hemoglobin A1c (HbA1c), aspartate aminotransferase (AST), alanine aminotransferase (ALT), gamma-glutamyl transferase, and high-sensitivity C-reactive protein (hsCRP); and a higher 10-year Framingham risk score (FRS), 10-year atherosclerotic cardiovascular disease (ASCVD) risk score, baseline CAC score, and last follow-up CAC score. In addition, the percentages of individuals with T2DM, hypertension, overweight, obesity, and metabolic syndrome were higher in the group with than in that without NAFLD. Comparison of the two groups of subjects with NAFLD showed that all these parameters were higher in subjects with intermediate/high than low FIB-4 scores. There were no significant intergroup differences in family history of T2DM, total cholesterol concentration, or follow-up interval.

**Table 1:** Baseline characteristics of the study population according to the baseline NAFLD status and liver fibrosis severity based on the FIB-4 score.

	Total	Non-NAFLD	NAFLD		*P*
			Low	Intermediate/high	
N (%)	1173	629 (53.6)	374 (31.9)	170 (14.5)	
Age (years)*	54.1 ± 7.4	54.3 ± 7.6	52.7 ± 7.5	56.5 ± 5.9	< 0.001
Male (n, %)	956 (81.5)	462 (73.4)	337 (90.1)	157 (92.4)	< 0.001
BMI (kg/m^2^)*	25.0 ± 3.0	23.9 ± 3.0	26.0 ± 2.7^a^	26.4 ± 2.5^a^	< 0.001
WC (cm)*	87.0 ± 8.2	83.8 ± 7.9	90.1 ± 6.9	91.9 ± 6.8	< 0.001
Systolic BP (mmHg)*	119.5 ± 12.9	117.5 ± 12.9	121.2 ± 12.7^a^	123.1 ± 11.7^a^	< 0.001
Diastolic BP (mmHg)*	76.6 ± 10.6	74.9 ± 10.6	78.3 ± 10.5^a^	79.4 ± 9.8^a^	< 0.001
Current smoker (n, %)	321 (27.4)	149 (23.7)^a^	132 (35.3)	40 (23.5)^a^	< 0.001
Moderate drinker (n, %)	623 (53.1)	309 (49.1)^a^	212 (56.7)^b^	102 (60.0)^ab^	0.01
Physically active (n, %)	661 (56.4)	325 (51.7)^a^	236 (63.1)	100 (58.8)^a^	0.002
Family history of T2DM (n, %)	282 (24.0)	138 (21.9)	102 (27.3)	42 (24.7)	0.157
T2DM (n, %)	155 (13.2)	49 (7.8)^a^	69 (18.4)^b^	37 (21.8)^c^	< 0.001
Hypertension (n, %)	393 (33.5)	170 (27.0)	151 (40.4)^a^	72 (42.4)^a^	< 0.001
Overweight (n, %)	904 (77.1)	408 (64.9)	337 (90.1)^a^	159 (93.5)^a^	< 0.001
Obese (n, %)	571 (48.7)	209 (33.2)	242 (64.7)^a^	120 (70.6)^a^	< 0.001
Metabolic syndrome (n, %)	375 (32.0)	103 (16.4)	186 (49.7)^a^	86 (50.6)^a^	< 0.001
FPG (mg/dL)*	104.5 ± 18.5	100.3 ± 15.2	108.3 ± 21.5	111.5 ± 18.7	< 0.001
HbA1c (%)†	5.5 (5.3–5.9)	5.5 (5.2–5.7)	5.7 (5.4–6.0)^a^	5.7 (5.4–6.2)^a^	< 0.001
Total cholesterol (mg/dL)*	199.1 ± 32.0	198.3 ± 30.6	198.8 ± 34.2	202.5 ± 32.1	0.299
TG (mg/dL)†	116 (85–162)	96 (71–135)	136 (106–194)^a^	135 (106–193)^a^	< 0.001
LDL-C (mg/dL)*	126.0 ± 28.5	124.1 ± 27.6^a^	127.6 ± 29.9^b^	129.8 ± 28.0^ab^	0.026
HDL-C (mg/dL)*	52.0 ± 13.1	56.1 ± 13.6	46.8 ± 11.2^a^	48.5 ± 9.6^a^	< 0.001
Uric acid (mg/dL)*	5.8 ± 1.4	5.5 ± 1.3	6.3 ± 1.4^a^	6.1 ± 1.3^a^	< 0.001
AST (U/L)†	25 (22–31)	24 (21–29)	25 (22–31)	34 (28–42)	< 0.001
ALT (U/L)†	23 (17–31)	20 (15–25)	28 (20–37)^a^	30 (21–42)^a^	< 0.001
GGT (U/L)†	25 (17–40)	20 (14–32)	30 (21–43)^a^	35 (22–61)^a^	< 0.001
hsCRP (mg/dL)†	0.06 (00.3–0.13)	0.05 (0.03–0.11)	0.07 (0.04–0.15)^a^	0.08 (0.04–0.15)^a^	< 0.001
10-year FRS (%)†	6.0 (3.0–10.0)	6.0 (2.0–10.0)	8.0 (4.0–12.0)	10.0 (6.0–12.0)	< 0.001
10-year ASCVD risk score (%)†	5.5 (2.7–9.7)	4.5 (2.1–8.3)	5.9 (3.2–10.7)	8.3 (5.3–12.0)	< 0.001
Baseline CAC score†	0.0 (0.0–21.6)	0.0 (0.0–15.0)^a^	0.0 (0.0–21.8)^a^	4.6 (0.0–63.5)	< 0.001
0 (n, %)	677 (57.7)	393 (62.9)	212 (56.8)	72 (42.6)	
1–100 (n, %)	357 (30.4)	175 (28.0)	112 (30.0)	707 (41.4)	
101–300 (n, %)	84 (7.2)	30 (4.8)	35 (9.4)	19 (11.2)	
> 300 (n, %)	49 (4.2)	27 (4.3)	14 (3.8)	8 (4.7)	
Last follow-up CAC score†	0.0 (0.0–47.9)	0.0 (0.0–36.5)^a^	1.2 (0.0–47.6)^a^	23.2 (0.0–113.5)	< 0.001
0 (n, %)	583 (49.7)	349 (55.8)	174 (46.8)	60 (35.3)	
1–100 (n, %)	384 (32.7)	193 (30.9)	128 (34.4)	63 (37.1)	
101–300 (n, %)	122 (10.4)	50 (8.0)	44 (11.8)	28 (16.5)	
> 300 (n, %)	78 (6.6)	33 (5.3)	26 (7.0)	19 (11.2)	
Follow-up interval (years)†	3.0 (2.0–3.8)	2.9 (2.0–3.8)	2.9 (2.0–3.8)	2.9 (2.0–3.7)	0.697

*Data are expressed as mean ± standard deviation.

^†^Data are expressed as median (interquartile range).

^a, b^The same letters indicate a statistically insignificant difference.

*P* value is for three groups.

ALT, alanine aminotransferase; ASCVD, atherosclerotic cardiovascular disease; AST, aspartate aminotransferase; BMI, body mass index; BP, blood pressure; CAC, coronary artery calcification; FIB-4, fibrosis-4 index; FPG, fasting plasma glucose; GGT, gamma-glutamyl transferase; HbA1c, hemoglobin A1c; HDL-C, high-density lipoprotein cholesterol; hsCRP, high-sensitivity C-reactive protein; LDL-C, low-density lipoprotein cholesterol; N, number; T2DM, type 2 diabetes mellitus; TG, triglyceride; WC, waist circumference.

### Association between liver fibrosis based on the FIB-4 score and baseline CAC score

**Figure 1:**
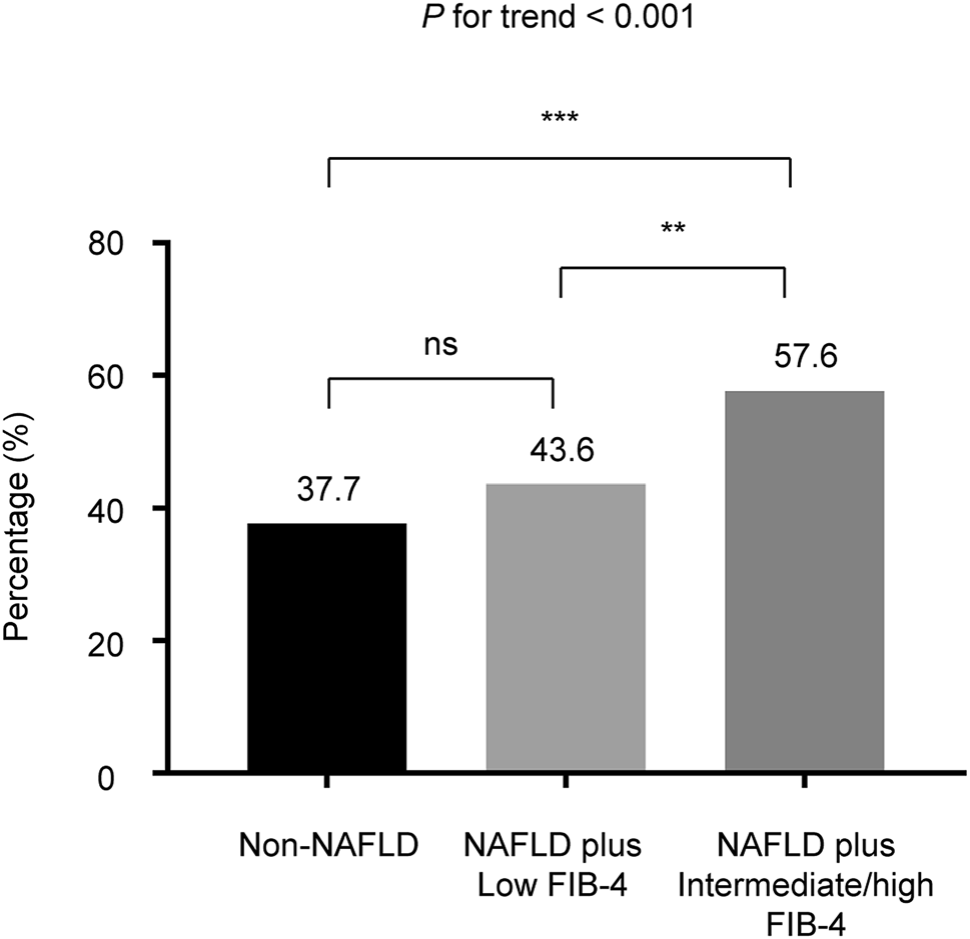
Proportion of subjects with a baseline coronary artery calcification score > 0 according to the baseline NAFLD status and liver fibrosis severity based on the FIB-4 score. ***P < 0.001, **P < 0.01. FIB-4, fibrosis-4 index; NAFLD, nonalcoholic fatty liver disease.

Of the study population, 42.2% had baseline CAC scores > 0, with the proportion of subjects with a positive baseline CAC score being significantly higher in the group with NAFLD plus intermediate/high FIB-4 scores (57.6%) than in the group without NAFLD (37.7%) and the group with NAFLD plus low FIB-4 scores (43.6%) (Fig. 1).

Multiple logistic regression analysis with the baseline CAC score as a dependent variable found that the odds ratio (OR) for CAC detection was significantly higher in subjects with NAFLD and intermediate/high FIB-4 scores than in subjects without NAFLD (OR, 2.27; 95% confidence interval [CI], 1.60–3.20; Table 2). This significance was no longer observed after adjustment for sex and BMI; smoking, drinking, and exercise habits; presence of hypertension and T2DM; and serum concentrations of triglycerides (TG), high-density lipoprotein cholesterol (HDL-C), low-density lipoprotein cholesterol (LDL-C), and hsCRP.

**Table 2:** Association between liver fibrosis severity based on the fibrosis-4 index score and baseline CAC score.

	Non-NAFLD	NAFLD	
		Low	Intermediate/high
Crude OR	1.00 (Ref)	1.28 (0.98–1.66)	2.27 (1.60–3.20)
Model 1	1.00 (Ref)	0.91 (0.68–1.20)	1.56 (1.08–2.25)
Model 2	1.00 (Ref)	0.82 (0.61–1.11)	1.41 (0.96–2.07)
Model 3	1.00 (Ref)	0.83 (0.61–1.13)	1.39 (0.94–2.05)

OR for CAC score > 0 in reference to a CAC score = 0.

Model 1 was adjusted for sex and body mass index.

Model 2 was adjusted for the variables included in model 1 plus smoking, drinking, and exercise habits; and the presence of hypertension and type 2 diabetes mellitus.

Model 3 was adjusted for the variables included in model 2 plus triglyceride, high-density lipoprotein cholesterol, low-density lipoprotein cholesterol, and high-sensitivity C-reactive protein concentrations.

CAC, coronary artery calcification; NAFLD, nonalcoholic fatty liver disease; OR, odds ratio.

### Association between liver fibrosis based on the FIB-4 score and CAC score progression

**Figure 2:**
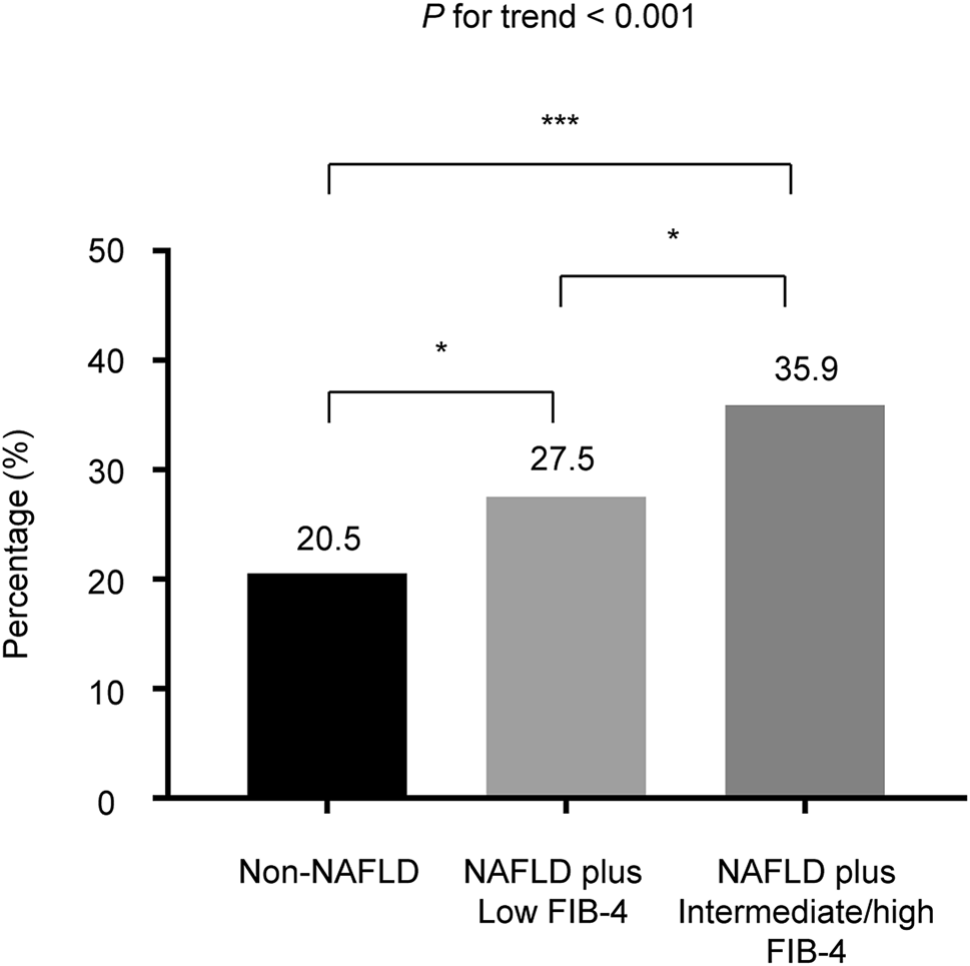
Proportion of subjects with coronary artery calcification score progression according to the baseline NAFLD status and liver fibrosis severity based on the FIB-4 score. ***P < 0.001, *P < 0.05. FIB-4, fibrosis-4 index; NAFLD, nonalcoholic fatty liver disease.

During the follow-up period, 35.9% (61/170) of subjects in the NAFLD plus intermediate/high FIB-4 score group, 27.5% (103/374) of subjects in NAFLD plus low FIB-4 score group, and 20.5% (129/629) of subjects in the non-NAFLD group showed CAC score progression (Fig. 2).

Multiple logistic regression analyses showed a graded association between liver fibrosis stage as assessed by the FIB-4 score and CAC score progression. Compared with that of subjects without NAFLD, the crude ORs for CAC progression were 1.47 (95% CI, 1.10–1.99) in subjects with NAFLD plus low FIB-4 scores and 2.17 (95% CI, 1.50–3.14) in subjects with NAFLD plus intermediate/high FIB-4 scores (Table 3). After adjustment for sex and BMI; smoking, drinking, and exercise habits; presence of hypertension and T2DM; and serum concentrations of TG, HDL-C, LDL-C, and hsCRP, the risk of CAC score progression remained significantly higher in subjects with NAFLD plus intermediate/high FIB-4 scores than in subjects without NAFLD (OR, 1.70; 95% CI, 1.12–2.58).

**Table 3:** Association between liver fibrosis severity based on the fibrosis-4 index score and progression of coronary artery calcification.

	Non-NAFLD	NAFLD	
		Low	Intermediate/high
Crude OR	1.00 (Ref)	1.47 (1.10–1.99)	2.17 (1.50–3.14)
Model 1	1.00 (Ref)	1.24 (0.90–1.70)	1.78 (1.21–2.62)
Model 2	1.00 (Ref)	1.17 (0.85–1.62)	1.73 (1.16–2.57)
Model 3	1.00 (Ref)	1.16 (0.82–1.64)	1.70 (1.12–2.58)

Model 1 was adjusted for sex and body mass index.

Model 2 was adjusted for the variables included in model 1 plus smoking, drinking, and exercise habits and the presence of hypertension and type 2 diabetes mellitus.

Model 3 was adjusted for the variables included in model 2 plus baseline coronary artery calcification score, follow-up interval, and triglyceride, high-density lipoprotein cholesterol, low-density lipoprotein cholesterol, and high-sensitivity C-reactive protein concentrations.

NAFLD, nonalcoholic fatty liver disease; OR, odds ratio.

Similar results were observed in the sensitivity analysis using the NFS instead of the FIB-4 score. Compared with that of subjects without NAFLD, the ORs for CAC progression in subjects with NAFLD plus intermediate/high NFS were 1.98 (95% CI, 1.33–2.96) in the unadjusted model and 1.57 (95% CI, 1.02–2.44) in the adjusted model (Supplementary Information Table 1).

## Discussion

The present study evaluated the association between liver fibrosis assessed using noninvasive fibrosis markers and CAC progression in subjects with NAFLD. We observed that CAC progression occurred more frequently in the group with NAFLD and higher fibrosis scores than in the group without NAFLD. In addition, baseline NAFLD and noninvasively assessed liver fibrosis stage were positively associated with the risk of CAC score progression. Individuals with NAFLD and a more advanced fibrosis stage with a higher FIB-4 score were at a significantly higher risk of CAC progression (OR, 1.70; 95% CI, 1.12–2.58) than individuals without NAFLD. Moreover, in the sensitivity analysis, similar results were obtained for the association between fibrosis stage stratified by the NFS and CAC progression.

Although the baseline CAC score measured by MDCT has been represented as a surrogate marker for CAC^17,18^, previous studies reported that CAC progression is significantly associated with incident cardiovascular events and mortality^15,18^. Because atherosclerosis progression is a dynamic and ongoing process, CAC progression may be a more effective predictor of future cardiovascular events than the baseline CAC score^19^. Therefore, here we evaluated CAC score progression using serial MDCT scans. Interestingly, we found that subjects with an advanced liver fibrosis stage determined using liver fibrosis markers were at a significantly higher risk for CAC progression after adjustment for known metabolic factors as confounders.

To our knowledge, the present study is the first to investigate the association between noninvasive liver fibrosis score and CAC progression. Advanced liver fibrosis stage assessed using a noninvasive fibrosis marker increased the risk of CAC progression in subjects with NAFLD. However, subjects with a low probability of liver fibrosis did not show an increased risk of CAC progression. Similarly, a long-term follow-up study of NAFLD with biopsy-proven fibrosis stage showed that subjects with advanced fibrosis were at an increased risk of CVD death (stage 3, 4; hazard ratio [HR], 1.55), whereas subjects at an early liver fibrosis stage were not^6^.

Although the mechanisms responsible for the association between liver fibrosis and CAC progression remain unclear, several possibilities have been suggested. Endothelial dysfunction triggered by persistent chronic inflammation and oxidative stress was shown to induce coronary atherosclerosis and liver fibrosis in patients with NAFLD/NASH^20,21^. NASH was also reportedly associated with prothrombotic factors^22^. This coagulation factor imbalance resulted in a positive link between CVD and liver fibrosis in subjects with NAFLD^4^. Moreover, pro-inflammatory cytokines were shown to induce abnormal lipid metabolism, chronic inflammation, and oxidative stress in subjects with NAFLD and liver fibrosis, suggesting that this pathogenic mechanism may be involved in the systemic inflammation that leads to CVD^21,23–25^. Pathophysiological evidence has helped establish a strong correlation between an emerging prevalence of NAFLD/NASH and an increased risk of CVD^26^. Thus, therapeutic candidates based on the pathogenesis of NAFLD/NASH probably exert beneficial effects against CVD events^4,26^. In this respect, noninvasive biomarkers of liver fibrosis would have clinical value for assessing liver fibrosis severity and future CVD risk.

The present study found that the noninvasive assessment of liver fibrosis in subjects with NAFLD was not significantly associated with the baseline CAC score after adjustment for confounding factors. One possible explanation for this discrepancy is that our study included participants who underwent routine health check-ups and excluded those with a history of CVD. In two previous studies, patients at high risk of CVD (baseline CAC score > 100) has been reported to correlate with high liver fibrosis score in subjects with NAFLD^7,8^. In addition, a previous study reported that patients with CAC progression combined with moderate to severe CAC scores (> 100) have an increased the risk of all-cause mortality^16^. However, only 11.3% of subjects in the present study had a baseline CAC score > 100, and 57.7% had a baseline CAC score = 0. Therefore, the assessment of a low-risk population in our study may have reduced the association between noninvasive liver fibrosis markers and the baseline CAC score. However, a positive association was obtained between CAC score progression and liver fibrosis markers for the low-risk population in this study. Thus, CAC score progression, not baseline CAC, could be a good prognostic marker for assessing the correlation with noninvasive liver fibrosis score, even in low-risk populations. These results also suggest that biomarker-based liver fibrosis stage can predict long-term dynamic changes in coronary atherosclerosis, rather than the baseline CAC score.

In the present study, NAFLD was diagnosed by ultrasonography instead of liver biopsy. Although the overall sensitivity and specificity of ultrasonography are approximately 85% and 94%^27^, respectively, it is considered to have a relatively low sensitivity for small hepatic steatosis^28^. Therefore, the true incidence of NAFLD could be underestimated in our study. However, ultrasonography is a widely accessible imaging technique for the diagnosis of fatty liver owing to its high safety, noninvasive nature, low cost, and ease of use. Recent studies reported that ultrasonography has adequate accuracy for detecting hepatic steatosis in as little as 10–20% of the liver^29,30^. Therefore, the use of ultrasonography is reliable for diagnosing fatty liver and has relatively few limitations compared with biopsy.

Noninvasive fibrosis scoring systems including the FIB-4 score and NFS are widely used to identify liver fibrosis severity. These noninvasive fibrosis assessments yield a high sensitivity and negative predictive value but a low positive predictive value, suggesting that it is better to exclude than detect advanced liver fibrosis^13,31^. False positive results of the intermediate/high fibrosis stage assessed with the FIB-4 score or NFS could have occurred in the present study, possibly diluting the association between liver fibrosis score and CAC progression. However, previous data indicated that the NFS could be an effective biomarker for predicting cardiovascular risk and mortality^32,33^. Although other noninvasive diagnostic techniques for predicting liver fibrosis, such as ultrasound elastography, could enable a better estimation of liver fibrosis, this technique is not always available in clinical practice^31^. Thus, noninvasive fibrosis scoring systems have diagnostic efficacy for identifying liver fibrosis in patients with NAFLD.

This study had several limitations. First, our subjects were recruited during general health examinations, so they did not represent the general population and laboratory test of chronic liver disease were conducted only for hepatitis B and C. Therefore, we were unable to collect data for other etiologies of chronic liver disease (e.g., tests for antinuclear, antimitochondrial, smooth muscle, and liver kidney microsome type-1 antibodies). Second, patients at a high risk of CAC progression may have undergone repeated MDCT during follow-up; thus, there was a high prevalence of NAFLD and male patients. This might have further contributed to our cohort not representing the general population. Third, since an alcohol consumption history could not be obtained quantitatively, it was impossible to fully discriminate between alcoholic fatty liver disease and NAFLD. However, the relative contribution of alcohol consumption to the development of NAFLD is controversial^34^. Finally, information on lipid-lowering agents other than statins was not obtained; such other drugs may have affected the calcification in subjects with coronary atherosclerosis.

In conclusion, to our knowledge, the present study is the first to show that advanced liver fibrosis stage assessed using a noninvasive fibrosis marker is an independent and significant contributor to CAC progression in subjects with NAFLD. Our findings suggest that noninvasive assessment of liver fibrosis degree is a useful indicator for predicting an increased risk of the development of CVD among subjects with NAFLD.

## Methods

### Ethics statement

In accordance with the ethical guidelines of the declaration of Helsinki and Korea Good Clinical Practice, all subjects provided written informed consent, and this study was approved by the institutional review board of Asan Medical Center (No. 2020-0343).

### Study population

A total of 7300 subjects underwent baseline coronary computed tomography angiography (CCTA) using MDCT scan during general health check-ups at the Health Screening and Promotion Center of Asan Medical Center (Seoul, Republic of Korea) in 2007–2011. The follow-up examinations for each subject were evaluated. Of them, 1591 subjects underwent repeat CCTA until December 2014. Subjects were excluded if they were treated with statins (n = 238); had a history of CVD (n = 95), percutaneous coronary artery procedure (n = 8), or coronary artery surgery (n = 3); were positive for hepatitis B (n = 48), hepatitis C (n = 19), or hepatocellular carcinoma (n = 4); or were liver transplant recipients (n = 2). Several subjects met ≥ 2 exclusion criteria. Finally, a total of 1173 subjects were analyzed.

Each subject completed a questionnaire addressing medications, previous medical or surgical history, and drinking and smoking habits. Drinking habits were classified based on frequency, with once or twice weekly considered moderate; smoking habits were classified as non-current or current; and exercise habits were classified based on frequency, with two or three times weekly considered physically active^35^. A history of CVD was defined as physician-diagnosed angina, myocardial infarction, and/or cerebrovascular accidents. T2DM was defined as an FPG ≥ 126 mg/dL and/or HbA1c concentration ≥ 6.5% and/or the use of anti-diabetic medications. Hypertension was defined as a systolic and/or diastolic BP ≥ 140/90 mmHg or the use of anti-hypertensive medications. Cardiovascular risk was determined by calculating 10-year FRS and 10-year ASCVD risk scores^36^.

### Definitions of NAFLD and the liver fibrosis score

NAFLD was diagnosed on hepatic ultrasonography (Ultrasound Systems IU22; Philips, Holland) by expert radiologists who were unaware of the patients’ health data. Fatty liver was diagnosed according to characteristic ultrasonographic findings, such as parenchymal brightness, liver-to-kidney contrast, blurring vessel, focal fat sparing, and narrowing of the hepatic vein lumen^37–39^.

Liver fibrosis severity in patients with NAFLD was determined using two noninvasive markers of liver fibrosis: the FIB-4 score and the NFS. The FIB-4 score, which has been validated for assessing the fibrosis stage in patients with NAFLD, was calculated as follows: FIB-4 score = (age [years] × AST [U/L])/(platelet count [× 10^9^/L] × ALT [U/L]^1/2^). Subjects were categorized into three groups as follows: those aged < 65 years with low (< 1.30), intermediate (1.30–2.66), and high (≥ 2.67) FIB-4 scores^40,41^ and those aged ≥ 65 years with low (< 2.00), intermediate (2.00–2.66), and high (≥ 2.67) FIB-4 scores^41^. For the sensitivity analysis, the NFS was calculated using the following formula: NFS = –1.675 + 0.037 × age (years) + 0.094 × BMI (kg/m^2^) + 1.13 × impaired fasting glucose or T2DM (yes = 1, no = 0) + 0.99 × AST/ALT ratio – 0.013 × platelet (× 10^9^/L) − 0.66 × albumin (g/dL). Subjects were categorized into three NFS groups as follows: those aged < 65 years with low (< − 1.455), intermediate (0.676 to − 1.455), and high (> 0.676) probabilities of advanced fibrosis^12,41^ and those aged ≥ 65 years with low (< 0.120), intermediate (0.120–0.676), and high (> 0.676) probabilities of advanced fibrosis^41^.

### Measurement of the CAC score

CAC scores were assessed by MDCT performed using a 64-slice single-source (LightSpeed VCT; GE, Milwaukee, WI, USA) or dual-source (Somatom Definition or Somatom Definition Flash; Siemens, Erlangen, Germany) CT device^42^. The CAC score was calculated using the Agatston scoring method^11^, and subjects with scores of 0, 1–100, 101–300, and > 300 were categorized as none, mild, moderate to severe, and severe, respectively^43^.

CAC progression was defined as: incident CAC, as indicated by a baseline Agatston score of 0 and a higher score on a follow-up examination^44^; or a baseline score > 0 and a ≥ 2.5-unit increase from the baseline to the final CAC score square root^16,45^. Prior to the determination of CAC progression, the CAC score square root was calculated to reduce dependence on residual interscan variability.

### Statistical analysis

Continuous variables with normal or skewed distributions are expressed as mean (standard deviation) or mean (interquartile range), respectively. Categorical variables are expressed as frequencies and percentages. The baseline data of the subgroups categorized by NAFLD and liver fibrosis stage were compared by one-way analysis of variance with Scheffe’s methods, the Kruskal–Wallis test, or χ^2^ tests. Multiple logistic regression analyses were conducted to evaluate the ORs and 95% CIs of the subgroups defined by the NAFLD status and liver fibrosis severity based on the FIB-4 score, relative to the baseline and CAC progression. All statistical analyses were performed with the SPSS software version 21.0 for Windows (IBM, Inc., Armonk, NY, USA). Values of *P* < 0.05 were considered statistically significant.

## Supplementary information

Supplementary Information

## Data Availability

The datasets generated and/or analyzed during the current study are available from the corresponding author on reasonable request.
