# Supplementary material for: Association between noninvasive assessment of liver fibrosis and coronary artery calcification progression in patients with nonalcoholic fatty liver disease
Source: Sci Rep. 2020 Oct 27;10:18323. doi: 10.1038/s41598-020-75266-4 (PMC7591518; doi:10.1038/s41598-020-75266-4)
Supplement: Supplementary file 1 — Supplementary Information [file 41598_2020_75266_MOESM1_ESM.docx]

**Supplementary appendix**

**Association between Noninvasive Assessment of Liver Fibrosis and Coronary Artery Calcification Progression in Patients with Nonalcoholic Fatty Liver Disease**

Jiwoo Lee, Hwi Seung Kim, Yun Kyung Cho, Eun Hee Kim, Min Jung Lee, In Yong Bae, Chang Hee Jung, Joong-Yeol Park, Hong-Kyu Kim, Woo Je Lee

**Supplementary Table 1.** Association between liver fibrosis severity based on the NFS and coronary artery calcification progression.

|  | Non-NAFLD | NAFLD | |
| --- | --- | --- | --- |
|  |  | Low | Intermediate/High |
| Crude OR | 1.00 (Ref) | 1.58 (1.18–2.10) | 1.98 (1.33–2.96) |
| Model 1 | 1.00 (Ref) | 1.37 (1.02–1.85) | 1.71 (1.14–2.56) |
| Model 2 | 1.00 (Ref) | 1.36 (1.01–1.84) | 1.60 (1.06–2.42) |
| Model 3 | 1.00 (Ref) | 1.30 (0.94–1.81) | 1.57 (1.02–2.44) |

Model 1 was adjusted for sex.

Model 2 was adjusted for the variables included in model 1 plus smoking, drinking, and exercise habits, and the presence of hypertension.

Model 3 was adjusted for the variables included in model 2 plus baseline coronary artery calcification score, follow-up interval, and triglyceride, high-density lipoprotein cholesterol, low-density lipoprotein cholesterol, and high-sensitivity C-reactive protein concentrations.

NAFLD, nonalcoholic fatty liver disease; OR, odds ratio; NFS, NAFLD fibrosis score
